# Ocean acidification may be increasing the intensity of lightning over the oceans

**DOI:** 10.1038/s41598-020-79066-8

**Published:** 2020-12-14

**Authors:** Mustafa Asfur, Jacob Silverman, Colin Price

**Affiliations:** 1 Faculty of Marine Sciences, Ruppin Academic Center, Mikhmoret, Israel; 2 National Institute of Oceanography (IOLR), Tel Shikmona, Haifa, Israel; 3 Porter School of the Environment and Earth Sciences, Tel Aviv University, Tel Aviv, Israel

**Keywords:** Climate sciences, Ocean sciences

## Abstract

The anthropogenic increase in atmospheric CO_2_ is not only considered to drive global warming, but also ocean acidification. Previous studies have shown that acidification will affect many aspects of biogenic carbon uptake and release in the surface water of the oceans. In this report we present a potential novel impact of acidification on the flash intensity of lightning discharged into the oceans. Our experimental results show that a decrease in ocean pH corresponding to the predicted increase in atmospheric CO_2_ according to the IPCC RCP 8.5 worst case emission scenario, may increase the intensity of lightning discharged into seawater by approximately 30 ± 7% by the end of the twenty-first century relative to 2000.

## Introduction

Global thunderstorms produce an estimated 10–14 cloud-to-ground lightning flashes every second, with most of this activity occurring in the tropics^1^. Physical models of lightning based on electrodynamic theory have considered the geometry of the ground into which the lightning is discharged, but not the composition/electrical characteristics of the ground implicitly, in their equations^2,3^. Perhaps as a result of this treatment, most meteorological/climate models including lightning have considered only the physical processes responsible for charge formation, distribution and accumulation and its eventual discharge from clouds^4–7^.

Such studies generally conclude that lightning flash frequency will increase in the future^6,8–10^, although one recent study actually points to a possible decrease in lightning activity^11^ as a result of global warming.

In a recent report, Asfur et al*.*^12^ demonstrated experimentally for the first time that the Lightning Flash Intensity (LFI) generated in a laboratory setup is positively correlated with the amount of salts dissolved in the water into which it is discharged. Where, the LFI is the integrated emission spectra measured by an optical sensor in the wavelength range of 150–1050 nm of the gases that are ionized by a laboratory generated electrical spark. This experimental outcome was used to demonstrate the positive effect that seawater salinity has on LFI that could be used to partially explain the higher intensity of lightning observed over the oceans in comparison to land^13–19^. The cited studies proposed that the difference between the intensities of cloud-to-ground lightning discharges over land and sea could arise from differences in updraft currents, charge buildup and distributions in thunderstorm clouds. Clearly, the experimental results of Asfur et al*.*^12^ are most likely explained by the higher conductivity of seawater in comparison to soils (*0*10^3^ greater). Thus, other physicochemical properties of natural waters that effect their conductivity, could also influence the LFI. One such property, which is known to increase the conductivity of dilute solutions is their acidity^20^. In this study, we examine the effects of seawater acidity on the LFI in natural seawater from the Mediterranean Sea before and after adjusting their pH by addition of a strong acid and by the bubbling of CO_2_ gas. The latter is a particularly relevant issue to consider in the context of global change processes, specifically with respect to ocean acidification that is occurring as a result of the anthropogenic increase in atmospheric CO_2_^21^. Where, excess atmospheric CO_2_ is absorbed in seawater at the surface of the oceans, producing carbonic acid and decreasing pH. Furthermore, the decrease in pH causes a shift in speciation of dissolved inorganic carbon expressed by the following stoichiometric equation:

$$ {\text{CO}}_{2} + {\text{H}}_{2} {\text{O}} + {\text{CO}}_{3}^{ - 2} \to 2{\text{HCO}}_{3}^{ - } $$

Over the last three decades the pH of seawater measured in the surface layer at seven oceanic time series stations throughout the Atlantic and Pacific oceans in the northern hemisphere displayed decreasing trends in pH of ca. − 0.0013 ± 0.0003 to − 0.0026 ± 0.0006/year^22^, which as expected are well correlated with the increasing trend in atmospheric CO_2_ (+ 2–3 ppmv/year).

## Experimental design and measurements

LFI measurements were performed using the same experimental setup described in Asfur et al.^12^. In brief, electrical sparks into the seawater below were generated with a Boost Step-up power converter between an electrode suspended above the surface of the water (~ 1 cm) and a submerged electrode (~ 3 cm). The spark was generated by an electronic circuit that converts a low input voltage of 3–4 V to 1000 kV (~ 1 MV) using a power supply (LE305A 5 A 0–30 V) that provided a stable DC input current of 2 A and a Boost Stepup power Module with a maximum pulse output DC current of 0.5 A. In comparison, the electrostatic potential measured during thunderstorms is ~ *0*10^1^–10^2^ MV^23^ and a peak current electrical discharge that typically varies from several to a few hundred kiloamperes^18^. Upon activation of the external power supply, electrical sparks were continuously discharged into the water and their emission spectra were measured using an optical fiber spectrometer (OCEAN FX Spectrometer) that reports the emission per wavelength in Relative Irradiance Units (RIU) in the wavelength range of 150–1050 nm. The spectral data were collected and visualized using the Ocean Optics OceanView 1.6.7 A/D interface and software at an acquisition frequency of 100 kHz. We report here the integrated value of LFI over the entire spectral range in RIUs. We use the LFI (integrated optical emission spectra) of the generated spark, which in essence is due to the ionization of the gases in the path of the spark. In natural lightning it was shown that the emission spectra are well correlated with the temperature of the return stroke^24^ and the electric field^25^. Thus, it seems reasonable that the optical intensity of the generated spark can also be correlated with its electric field and therefore LFI is equivalent to its current as previously demonstrated by Wang et al*.*^26^. It should also be noted, but perhaps not too surprising, that the emission spectra peaks in our experiments^12^ coincide with those of field-based observations of natural lightning flashes^27^. However, there is a huge difference in peak current between the laboratory generated sparks and natural lightning and it remains to be shown that the experimental results can be extrapolated to nature.

In our experiments we measured the intensity of the generated sparks into freshly sampled and filtered (filtration through 0.45 µm GFF) Mediterranean seawater with an approximate salinity of 39 PSU, an initial pH of ca. 8.2 and total alkalinity of ca. 2600 µmole/kg, at room temperature of 20 °C. Detailed experimental conditions and results can be provided by the corresponding author upon request.

Initially, we adjusted the pH of the seawater (1.4 L) by incremental additions (25–50 µL) of a strong acid (~ 10% HCl). After each addition of acid the pH, temperature and conductivity of the solution were measured with a handheld WTW multi-meter (MultiLine Multi 3620 IDS). The pH was measured with a combination glass electrode and calibrated immediately before the experiments with WTW buffer solutions (pH 4.01 ± 0.02 and 7.00 ± 0.03). The specified precision of the electrode is ± 0.004 pH units. The pH values that were tested by strong acid additions were in the range 4.8–8.2 at 25 °C (initial value of ca. pH 8.2). In the CO_2_ bubbling experiments, after measuring the LFI of the initial seawater solution, we started to gently bubble for short periods (< 2 s) CO_2_ gas (99.999%) to produce carbonic acid that caused a reduction in pH from its initial value of ca. pH 8.2. After each bubbling period, the solution was gently stirred to homogenize it and pH was measured simultaneously until kinetic equilibrium was deemed to be attained when ΔpH/Δt <  + 0.001/5 s. The pH values that were tested by CO_2_ bubbling addition were in the range (5.6–8.2 at 25 °C). After the pH was deemed stable for each addition (acid and CO_2_) the power supply was turned on and the LFI of the generated sparks were repeatedly measured (ca. 8–10 times) over a period of 1–2 min. Water samples were taken at the beginning and end of each experiment for making measurements of total alkalinity (TA), pH and density in the laboratory. Analyses of TA samples were done with a Methrom 785 Titrino Plus potentiometric titration system with ~ 0.05 N HCl, based on the analytical procedures and calculations described by Sass and Ben-Yaakov^28^. TA measurements were calibrated and standardized using seawater CRMs from A. Dickson’s lab^29^. Laboratory seawater density measurements were made with a 6 digit accuracy Anton Paar DMA-5000 densitometer and converted to salinity values with the measurement temperature using the equation of state for seawater from the 19^th^ edition of standard methods^30^. pH samples were measured with a spectrophotometric system (total hydrogen scale) coupled with pH sensitive indicator dye m-Cresol purple (mCP), using the CONTROS HydroFIA-pH analyzer by Kongsberg-Contros^31^ with a specified accuracy of ± 0.003. The carbonate system parameters (HCO_3_^−^, CO_3_^−2^ and pCO_2_) presented in the results section were calculated with the CO2sys2.1.xls spreadsheet^32^ using the WTW pH and temperature measurements, density-derived salinity, measured TA and calculated intermediate values (for HCl treatments). In these calculations we employed the thermodynamic dissociation constants (K1 and K2) of Mehrbach et al*.*^33^, the hydrogen sulfate dissociation constant (KHSO_4_) of Dickson and Millero^34^ and the salinity-derived total borate value of Lee et al*.*^35^.

## Results

**Figure 1:**
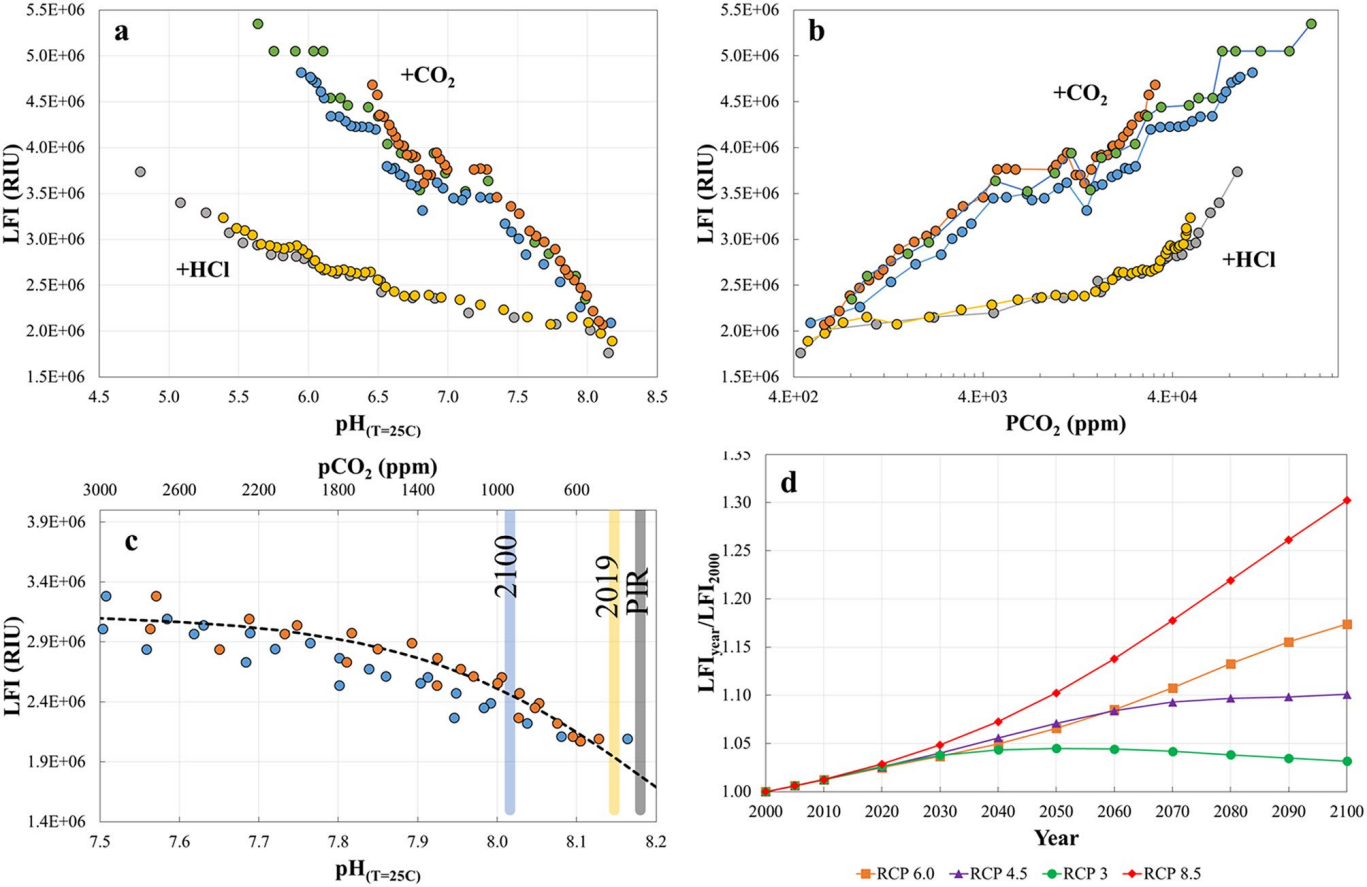
(**a**) Dependence of LFI on seawater pH adjusted to a constant temperature of 25 °C, and (**b**) calculated values of pCO_2_ (calculated from pH and total alkalinity measurements at 25 °C), and (**c**) Change in LFI with pH and pCO_2_ from their pre-industrial levels (PIR in the plot) up to pCO_2_ = 3000 ppm and pH = 7.5, and (**d**) the ratio of calculated LFIs as a function of predicted atmospheric pCO_2_ according to RCP 3, 4.5, 6.0 and 8.5 compared to LFI in 2000 at 25 °C. In panels **a** and **b**, the different colored markers correspond to different experiments and treatments indicated by + HCl (addition of strong acid experiments) and + CO_2_ (CO_2_ bubbling experiments). In addition each data point represents an average of 8–10 repeated measurements of the emission spectra with a standard deviation of ± 4% (maximum). In panel **c** all data from the 3 CO_2_ bubbling experiments in the relevant range are included and the black dashed curve indicates the best fit of the logistic model (Eq. 1) to the LFI and corresponding pCO_2_ data (n = 24).

In general, our experimental results show that the LFI increases approximately linearly with decreasing seawater pH in response to both acid addition and CO_2_ bubbling (Fig. 1a). However, perhaps unexpectedly, the rate of increase in LFI with decreasing pH in the CO_2_ bubbling treatment is greater by a factor of 2.6 than in the strong acid addition. While in both cases, pH decreases or H^+^ ion concentrations increase with the addition of acids (+ HCl and + CO_2_ or carbonic acid), the alkalinity of the seawater decreases in response to + HCl, but does not change in the + CO_2_ treatments. Therefore, it is highly likely that the alkalinity of the solution also plays a role in the intensity of the electrical discharges (LFI).

Comparison of the LFI to the calculated seawater pCO_2_, also yielded a stronger response in the CO_2_ bubbling treatment compared to the strong acid treatment up to ca. 5000 ppm at a pH of ca. 7.0 (Fig. 1b). Above 5000 ppm, LFI increases at a greater rate in the acid addition treatment relative to the CO_2_ bubbling, but does not become higher within the experimental range. Interestingly, these changes occur when the CO_3_^−2^ ion concentration is vanishingly small in comparison to the HCO_3_^−^ ion concentration in the bubbling treatment and the Cl^−^ concentration in the acid addition treatment. It is possible that the addition of acid and bubbling of CO_2_ in seawater affect the inorganic speciation of ions dissolved in seawater leading to these differences in conductive properties of the solution and LFI^36^. Decreasing pH, whether caused by addition of acid or CO_2_ bubbling, leads to a reduction in CO_3_^−2^ and increase in HCO_3_^−1^ concentrations (see stoichiometric equation above for example).

In Fig. 1c we calculate the dependence of LFI on pCO_2_ and pH from the experimental results within ranges of climatic relevance (pH 7.5–8.2 at 25 °C; pCO_2_ = 200–2700 ppm). Within these ranges LFI increase by a factor of 1.5, which is considered a significant effect. With respect to the conditions in the recent past and not far off future, between the pre-industrial revolution (PIR) level of atmospheric pCO_2_ (280 ppm) and the present level (410 ppm), LFI increases by 10%, while between PIR and pCO_2_ = 1000 ppm, which is predicted to occur early in the next century according to RCP8.5 (ca. 2100), LFI increases by ca. 40%.

Finally, we calculate the LFI as a function of seawater pCO_2_ in equilibrium with the atmospheric partial pressure (Fig. 1d) at a temperature of 25 °C, predicted for the different emission scenarios between 2000 and 2100^37^. The lines in Fig. 1d are calculated from the best fit of the logistic model (Eq. 1) to the data presented in Fig. 1c, where c = 3,100,000 ± 85,000, a = 0.002 ± 0.0003 and b = 100 ± 90 (± 1STD; n = 24; r^2^ = 0.92; *p* < 0.0001).

1 $${LFI}_{\left(PC{O}_{2}\right)}=\frac{c}{\left(1+Exp\left(-a\cdot \left({pCO}_{2}-b\right)\right)\right)}$$

According to this model, LFI will increase by approximately 30 ± 7% between the years 2000 and 2100 in the worst case scenario where atmospheric pCO_2_ will continue to increase until 2100 (RCP 8.5 and 6.0). While in the stabilization and decreasing trends emission scenarios (RCP 4.5 and 3.0) LFI will increase to as much as 3 ± 1–10 ± 2% above the 2000 intensity by the end of the century.

## Summary and conclusions

Our experimental results show that the flash intensity of laboratory-generated electrical sparks has a strong positive dependence on the acidity or a negative correlation with the pH of the seawater into which it is discharged. It is highly likely that these results also apply to the natural environment. If this is indeed the case then the ongoing process of ocean acidification may cause an increase in the intensity of lightning discharged into seawater. Nonetheless, based on our results we predict a 30% increase in LFI for the worst case RCP 8.5 CO_2_ emission scenario due to ocean acidification alone. It should be noted that this prediction is based only on the relations developed for pCO_2_ and LFI at a constant temperature of 25 °C in seawater with a salinity of 39 PSU, and does not consider changes in the physical conditions affecting the electrical activity in thunderstorm clouds that may also change in response to global climate change^6,9,10^.

In Asfur et al*.*^12^ it was shown that LFI increases by a factor of 1.6 ± 0.3 per 1 mg/L increase in NaCl concentration, which is roughly equivalent to 1PSU (Practical Salinity Unit). The salinity range of surface seawaters is 30–40 PSU^38^ and its conductivity in this range at a temperature of 25 °C increases according to the equations of Fofonoff and Millard^30^ by ~ 2–3% per 1PSU from 46.2 to 59.7 mS/cm. Thus, assuming that the increase in LFI occurs in response to the change in conductivity of seawater, which is also affected by temperature^39^, it may be inferred that seawater warming that also increases conductivity, would also result in increased LFI. At a constant salinity of 35PSU, the conductivity increases in the range 10–30 °C from 38.1 to 58.4 mS/cm, or approximately + 2%/°C. In conclusion, our results suggest that current and future ocean acidification is a positive feedback that may potentially increase the intensity of lightning discharged into the oceans by the end of twenty-first century.
